# Effect of age, sex, and season on the variation in blood analytes of a clinically normal ex situ population of killer whales (*Orcinus orca*)

**DOI:** 10.1111/vcp.12697

**Published:** 2019-01-24

**Authors:** Hendrik H. Nollens, Todd R. Robeck, Todd L. Schmitt, Lara L. Croft, Steve Osborn, James F. McBain

**Affiliations:** ^1^ Veterinary Services SeaWorld San Diego San Diego California; ^2^ SeaWorld and Busch Gardens Species Preservation Laboratory SeaWorld Parks and Entertainment San Diego California; ^3^ Veterinary Services SeaWorld Orlando Orlando Florida; ^4^ Veterinary Services SeaWorld San Antonio San Antonio Texas

**Keywords:** aging, cetacean, Delphinidae, hematology, orca, serum biochemistry

## Abstract

**Background:**

The effects of sex, age, and season on blood analyte concentrations have not been investigated for the killer whale (*Orcinus orca*). Defining these changes provides background data for improving the care of managed populations and defines normal changes that could occur in wild counterparts.

**Objectives:**

We aimed to define hematologic and serum biochemical variation by age, sex, and season for an ex situ killer whale population.

**Methods:**

Blood samples collected from killer whales during normal wellness exams were retrospectively identified. Killer whales were categorized by age; calf (0‐2.9 years), juvenile (3‐10.9 years), early adult (11‐20.9 years), adult (21‐30.9 years), and aged (>30.9 years); sex; and season. Standard CBC and biochemistry were collated, and only samples without evidence of disease were used. A mixed effects maximum likelihood regression with animal identification (ID) as the random effects variable was used to compare groups with a significance set at *P* ≤ 0.01.

**Results:**

All analytes differed by age, while only four differed by sex. Red blood cell parameters and associated renal analytes increased with age, while liver‐associated analytes and glucose decreased. Season affected 59% of the blood analytes.

**Conclusions:**

Aged killer whales showed strong evidence of altered physiology as compared with younger animals. Anemia did not develop with age as was observed in one bottlenose dolphin population. Observed decreases in renal function could be caused by chronic disease or dehydration. Decreases in immune function parameters suggest immune senescence. These results provide background data for evaluating the health of managed and free‐ranging killer whales.

## 1 INTRODUCTION

The analysis of blood analytes is an important tool in an individual's health assessment. Hematology and serum chemistry are used to diagnose and evaluate physiologic, reproductive, and pathologic conditions.^1^ Ideally, analyte levels for an individual are compared against historical values from the same individual, or more commonly against the distribution of values from a reference population. The expected values observed in an individual that deviate from the reference population provide diagnostic information that can aid in selecting individual management procedures. Aging, physiologic conditions, environmental factors, and diseases can all cause deviations in the enzyme, metabolite, and mineral contents of an individual.^1^, ^2^, ^3^ In many species, reference intervals (RIs) have been determined for specific age and sex categories, resulting in a more sensitive and accurate tool to evaluate physiology and diagnose disease, while also providing insights into the physiology of aging.^4^, ^5^, ^6^, ^7^

Cetaceans represent an animal group that comprises all dolphins, porpoises, and whales. A small number of studies have reported on the clinicopathologic analytes of cetaceans, and a subset investigated host and environmental influences on these analytes. However, these studies almost exclusively focused on bottlenose dolphins and beluga whales.^8^, ^9^, ^10^, ^11^, ^12^, ^13^, ^14^, ^15^, ^16^, ^17^, ^18^, ^19^, ^20^, ^21^ While variability was detected in these studies, no consistent drivers of the variabilities were detected across studies or species. Challenges and limitations, especially relating to earlier studies, included low numbers of animals and samples, unknown health histories, different laboratory methods, and no control measures to account for fasting status and serum quality.

The first study reporting on pathologic data from killer whales (*Orcinus orca*) dates back to 1979^22^; however, this study did not include hematology or biochemistry data. The first study reporting clinicopathologic RIs for killer whales was published in 1983, but samples were collected, and data were analyzed from only 14 whales, and the analytical methodologies used are, in most cases, no longer available.^23^ The ex situ killer whale population housed at the SeaWorld facilities provides a unique window into killer whale physiology, and clinicopathologic data collected from this population were recently used to describe physiologic changes which are observed during normal pregnancies (Robeck and Nollens, 2013).^24^ The results of managed population studies are particularly valuable because they allow for longer term follow‐up of individuals of known age, health, and reproductive and nutritional statuses. Using data from 2823 blood samples collected as part of the routine preventative medicine program from 32 killer whales over a 22‐year period, the main objectives of this study were (a) to describe normal hematologic and serum biochemical analyte variation in clinically healthy killer whales; (b) to examine the effects of age, sex, and season on this variation; and (c) to generate insights into biological changes associated with aging in killer whales.

## 2 MATERIALS AND METHODS

Thirty‐two killer whales (*Orcinus orca*) were group housed in natural daylight habitats containing a minimum of 19 000 000 L natural or synthetic (Univar, Redmond, WA, USA) recirculating salt water that was maintained at approximately 14°C year‐round. Animals were fed a diet of frozen‐thawed whole fish which contained some or all of the following fish: Pacific herring (*Clupea harengus*), sardines (*Sardinops sagas*), Columbia River smelt (*Thaleichthys pacificus*), Pacific mackerel (*Scomber japonicas*), capelin (*Mallotus villosus*), and pink salmon (*Oncorhynchus gorbuscha*) at approximately 2%‐3% of their body weight per day. All food fish was suitable for human consumption. Animals were supplemented with Vita‐Zu Marine Mammal multivitamin tablets (Mazuri, St. Louis, MO, USA).

Fasting blood samples that were routinely collected and analyzed from all whales either bi‐weekly or monthly as part of SeaWorld's preventative medicine program between January 10, 1993 and October 16, 2013 provided the retrospective data for this study. To restrict the data to samples which had been collected from clinically healthy killer whales, samples drawn while on medication, other than multivitamin tablets, were excluded. For deceased whales, samples drawn within 30 days of death were excluded.^20^ Based on the previously demonstrated effects of gestation and lactation on hematologic parameters (Robeck and Nollens, 2013),^24^ all samples collected 18 months before and 6 months after parturition or while serum progesterone levels were elevated (>5000 pg/mL) for at least 5 weeks were excluded. Blood samples with any degree of hemolysis were excluded (Morgan et al, 1999).^25^

For venipuncture, the whales were trained to present the ventral surface of their fluke to the attending veterinarian for sampling using a 19‐gauge 1.5‐inch needle. Blood was collected into BD Vacutainer tubes (Becton Dickenson, Franklin Lakes, NJ, USA) containing potassium‐EDTA, sodium citrate, or thrombin. Hemoglobin concentration (Hb), red blood cell count (RBC), the mean corpuscular volume (MCV), red blood cell distribution width (RDW), platelet count (platelet), mean platelet volume (MPV), reticulocyte fraction (Retic), total white blood cell count (WBC), and segmented neutrophil (Abs segs), absolute lymphocyte (Abs lymphs), monocyte (Abs monos), and eosinophil counts (Abs eos), and erythrocyte sedimentation rates at 60 minutes (ESR 60) were measured using EDTA‐anticoagulated blood. Samples were processed using the SeaWorld on‐site diagnostic laboratory. The analytes Hb, RBC, MCV, RDW, platelet, MPV, Retic, and WBC were determined using either an Abbott Cell Dyn 3500R (Abbott Laboratories, Abbott Park, IL, USA) or Siemens Advia 2120i (Siemens Medical Solutions USA, Inc., Malvern, PA, USA) automated hematology analyzer using the manufacturer's reagents. From these analytes, hematocrit (HCT), mean corpuscular hemoglobin (MCH), and mean corpuscular hemoglobin concentration (MCHC) were calculated. The differential cell counts were derived by manually counting 100 cells in EDTA‐anticoagulated blood Wright‐Giemsa‐stained smears and then calculating the Abs segs, Abs lymphs, Abs monos, and Abs eos from the percentages multiplied by the WBCs. The biochemical parameters glucose, urea (BUN), creatinine (Creat), total bilirubin (Tbili), cholesterol, triglyceride, total protein (Tprotein), albumin, total globulins (globulin), alkaline phosphatase (ALP), alanine (ALT) and aspartate (AST) aminotransferases, gamma‐glutamyl transpeptidase (GGT), creatinine kinase (CK), lactate dehydrogenase (LDH), calcium (Ca), phosphorus (P), sodium (Na), potassium (K), chloride (Cl), CO_2_, and iron were measured using either the Ciba Corning Fast 4 (Ciba Corning, Cambridge, MA, USA) or the Olympus AU400E (Olympus Corporation, Center Valley, PA, USA) automated serum chemistry analyzer on thrombin‐coagulated serum. All reagents used were designed for and purchased from the manufacturer. Fibrinogen (FIB) content was quantified using the Organon Teknika Coag‐a‐mate (Organon Teknika Corporation, Durham, NC, USA) or the Sysmex CA‐500 (Sysmex America, Winter Springs, FL, USA) on citrate‐anticoagulated blood. The estimated sedimentation rate (ESR) 60 was determined using a Sediplast Westergren ESR system (LP Italiana SPA, Milan, Italy). Correlation studies were conducted when equipment was transitioned as part of the best laboratory practices program, by analyzing 30 blood or serum samples on both pieces of equipment and calculating the linear correlation coefficient (*R* ^2^) for each analyte (*R* ^2^ range 0.916‐0.995). The on‐site ASCP certified clinical laboratory scientists and field service engineers performed and documented analyzer calibrations and maintenance, and installed analyzer updates as prescribed by the manufacturer's recommended maintenance schedule to ensure instrument performance and reliability. On a regular basis, all clinical and analytical methods were tested with a multilevel, matched matrix, QC material at a minimum frequency as recommended by the assay manufacturer. QC test results were compared to establish RIs and validate assay performance.

For analysis, age at the time of sampling (in years) was calculated by subtracting the date of sampling by either the known or estimated birth date, divided by 365. For wild caught or stranded animals, a birth date of January 1st of the estimated birth year as estimated based on body length at capture (Robeck et al, 2015).^26^ Each sample was assigned to one of five age categories (coded 0‐4), defined as calf (0‐2.99 years), juvenile (3‐10.99 years), early adult (11‐20.99 years), adult (21‐30.99 years), and aged (>30.99 years). Samples were allocated by season (coded 0‐3) as follows: spring (March 1‐May 31), summer (June 1‐August 31), fall (September 1‐November 30), and winter (December 1‐February 29).

### 2.1 Statistical analyses

Statistical analyses were performed using Stata statistical software (version 14; StataCorp LP, College Station, TX, USA). A two‐stage mixed effects maximum likelihood (ML) regression model^27^, ^28^ (West et al.,2015, Robeck et al., 2017) quantifying the relationship between the dependent variable (analyte), the fixed effects variable (stage 1, animal age or age category, sex, and season) and the random effects variable, animal ID (stage 2, n = 11, coded 1‐32), was used to control for the variance associated with an unequal number of repeated measures per animal. The mixed effects model was used because it can incorporate the effect of the variance associated with the unbalanced design between and the correlated repeated measures from within each animal with the effects of any independent or fixed variables to predict their collective effect on the sample means (marginal means). The variation associated with the full model (all fixed and random effects) was initially determined, and then each fixed effects variable was removed iteratively in a backward direction. The two models were then compared (with and without the individual fixed effects variable that had been removed) using the LR test at a *P* < 0.05. The variable was then retained or omitted depending on whether it contributed significantly toward the model explanation (final model) of the dependent variable. For brevity, only fixed variables with significant effects were discussed in the results. All final mixed models were checked for normality using quantile plots of the standard residuals. If quantile‐quantile (qnorm) plots of standardized residuals exhibited non‐normal distributions, then data were transformed as predicted by the Shapiro‐Wilk test (Ladder command, STATA) until residuals were normalized. Finally, pairwise comparisons of the predicted marginal means between and within the fixed variables were made while applying the Šidák correction factor at a significance level of *P* < 0.01. For text and tables, any transformed data were first back‐transformed, and then all data were presented as marginal means with 95% confidence intervals (CI) unless noted otherwise.

## 3 RESULTS

The 32 killer whales consisted of 13 males (M) and 19 females (F). A total of 2823 blood samples met the inclusion criteria and were included in the study, resulting in 45 168 hematology and 64 929 serum chemistry data points. Ages at which blood samples were drawn ranged from 0.3 to 47.6 years with a respective mean and median of 14.2 and 11.8 years. The dataset consisted of 221 blood samples from calves (9 M & 7 F), 1,084 blood samples from juvenile whales (11 M &11 F), 794 blood samples from early adults (9 M & 11 F), 476 blood samples from adults (4 M & 9 F), and 248 blood samples from aged whales (1 M & 3 F). Seven hundred and one blood samples were collected in spring, 746 were collected in summer, 696 were collected in fall, and 680 were collected in winter.

Four (10%) of the analytes (RDW, MPV, triglycerides, and phosphorus) differed (*P* < 0.01) between the sexes (Table 1). Male killer whales had higher RDWs (mean ± SD: 14.06 ± 0.18 vs 13.30 ± 0.15), MPV (18.33 ± 0.55 vs 15.32 ± 0.53), triglycerides (149.1 ± 6.7 vs 127.9 ± 5.6), and phosphorus levels (6.00 ± 0.10 vs 5.69 ± 0.08) compared with female killer whales,.

**Table 1:** A list of 39 hematology and serum chemistry analytes measured in killer whales

Blood analyte	Sex	Age category	Season
Hematology			
Hb (g/dL)		+	+
HCT (%)		+	+
RBC (10^6^/μL)		+	+
MCV (fL)		+	+
MCH (pg)		+	
MCHC (g/dL)		+	
RDW (%)	+	+	
Platelet (10^3^/μL)		+	
MPV (fL)	+	+	
Retic (%)		+	+
WBC (10^3^/μL)		+	+
Abs segs (10^3^/μL)		+	+
Abs lymphs (10^3^/μL)		+	
Abs monos (10^3^/μL)		+	
Abs eos (10^3^/μL)		+	
ESR60 (mm/h)		+	
Serum chemistry			
Glucose (mg/dL)		+	+
BUN (mg/dL)		+	+
Creat (mg/dL)		+	+
TBili (mg/dL)		+	
Cholesterol (mg/dL)		+	+
Triglyceride (mg/dL)	+	+	+
TProtein (g/dL)		+	+
Albumin (g/dL)		+	+
Globulin (g/dL)		+	+
ALP (IU/L)		+	+
ALT (IU/L)		+	+
AST (IU/L)		+	+
GGT (IU/L)		+	+
CK (IU/L)		+	
LD (IU/L)		+	
Calcium (mg/dL)		+	
Phosphorus (mg/dL)	+	+	+
Sodium (mEq/L)		+	+
Potassium (mEq/L)		+	
Chloride (mEq/L)		+	+
CO_2_ (mEq/L)		+	+
Iron (μg/dL)		+	
FIB (mg/dL)		+	
N (%) analytes affected (*P *<* *0.05)	4 (10)	39 (100)	23 (59)

Hb, hemoglobin concentration; RBC, red blood cell count; MCV, mean corpuscular volume; RDW, red blood cell distribution width; platelet, platelet count; MPV, mean platelet volume; Retic, reticulocyte fraction; WBC, total white blood cell count; Abs segs, segmented neutrophil count; Abs lymphs, absolute lymphocyte count; Abs monos, monocyte count; Abs eos, eosinophil counts; ESR60, erythrocyte sedimentation rate at 60 minutes; BUN, blood urea nitrogen; Creat, creatinine; Tbili, total bilirubin; Tprotein, total protein; globulin, total globulins; ALP, alkaline phosphatase; ALT, alanine aminotransferase; AST, aspartate aminotransferase; GGT, gamma‐glutamyl transpeptidase; CK, creatinine kinase; LDH, lactate dehydrogenase; Ca, calcium; P, phosphorus; Na, sodium; K, potassium; Cl, chloride; FIB, fibrinogen.

Analytes for which sex, age, and season were a significant factor (*P* < 0.01) while correcting for each of the other factors, are indicated.

Differences (*P* < 0.01) were detected among at least some of the age categories for all 39 analytes (Tables 1 and 2). A progressive upward or downward trend with age category was detected in 33 (85%) analytes (Table 2). Hb, HCT, RBC, RDW, MPV, BUN, Creat, TBili, TProtein, Albumin, Globulin, and LD were progressively increased with age. MCH, MCHC, Platelet, Retic, WBC, Abs segs, Abs lymphs, Abs monos, Abs eos, ESR60, glucose, ALP, ALT, AST, CK, Calcium, Sodium, Potassium, CO_2_, iron, and FIB decreased progressively with age (Table 2). The number of differences (*P* < 0.01) detected among analytes of adjacent age groups also decreased with age, with the least differences detected between the adult and aged groups (17/39, 44%) (Table 2).

**Table 2:**
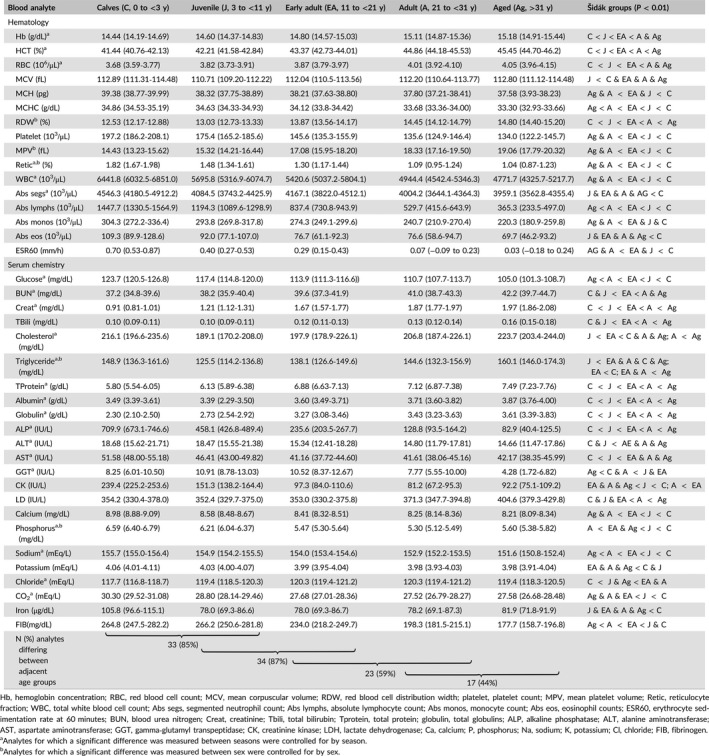
Hematologic and serum chemistry analytes (marginal mean, lower 95% confidence limit − higher 95% confidence limit) from 2823 blood samples of clinically healthy killer whales (*Orcinus orca*), controlling for sex and season

Seasonal differences (*P* < 0.01) were detected for 23 (59%) of the analytes (Tables 1 and 3, 4, 5, 6, 7). Seven (43%) hematology and 16 (70%) serum chemistry values differed between season. No seasonal differences (*P* > 0.01) were detected in MCH, MCHC, RDW, platelet count, MPV, Abs lymphs, Abs monos, Abs eos, ESR 60, TBili, CK, LD, calcium, potassium, iron, and FIB. When controlling for age groups, seasonal differences (*P* > 0.01) were no longer detected in MCV and globulins in any age group (Tables 3, 4, 5, 6, 7). Similarly, when controlling for age group, seasonal differences (*P* > 0.01) were no longer detected in the Hb levels of adult and aged whales.

**Table 3:** Hematologic and serum chemistry analytes (marginal mean, lower 95% confidence limit − higher 95% confidence limit) from 221 blood samples of clinically healthy killer whale (*Orcinus orca*) calves (0 to <3 years old) for which seasonal differences were detected (N = 23)

Blood analyte	Spring (Sp)	Summer (S)	Fall (F)	Winter (W)	Šidák groups (*P* < 0.01)
Hematology					
Hb (g/dL)	14.4 (14.2‐14.5)	14.6 (14.4‐14.8)	14.5 (14.3‐14.7)	14.3 (14.2‐14.5)	W & Sp < S
HCT (%)	41.3 (40.8‐41.8)	41.9 (41.3‐42.4)	41.5 (41.0‐42.0)	41.1 (40.1‐41.6)	W<F& Sp < S
RBC (10^6^/μL)	3.66 (3.57‐3.75)	3.71 (3.62‐3.80)	3.69 (3.60‐3.78)	3.64 (3.55‐3.74)	Sp & W < S & F
MCV (fL)	113.1 (111.88‐114.3)	113.0 (111.8‐114.2)	112.8 (111.5‐114.0)	112.7 (111.5‐113.9)	NSD
Retic^a^ (%)	1.91 (1.74‐2.07)	1.80 (1.64‐1.97)	1.80 (1.64‐1.97)	1.93 (1.77‐2.09)	S & F < Sp & W
WBC (10^3^/μL)	6509.4 (6093.6‐6925.1)	6375.8 (5959.6‐6792.0)	6342.4 (5925.3‐6759.5)	6545.8 (6128.7‐6962.8)	F < Sp & W; S<W
Abs segs (10^3^/μL)	4606.6 (4235.4‐4977.8)	4482.7 (4111.1‐4854.2)	4457.5 (4085.2‐4829.7)	4644.9 (4272.7‐5017.2)	F < Sp & W; S<W
Serum chemistry					
Glucose (mg/dL)	125.1 (121.9‐128.4)	119.8 (116.6‐123.1)	124.1 (120.8‐127.4)	125.9 (122.6‐129.2)	S<Sp & F & W; F<W
BUN (mg/dL)	36.3 (33.9‐38.7)	38.4 (36.0‐40.7)	37.6 (35.2‐40.0)	36.5 (34.1‐38.9)	Sp & W < F < S
Creat (mg/dL)	0.93 (0.83‐1.03)	0.94 (0.84‐1.04)	0.91 (0.80‐1.00)	0.85 (0.75‐0.96)	W < Sp & S & F
Cholesterol (mg/dL)	212.9 (193.2‐232.6)	219.6 (199.9‐239.2)	217.5 (197.8‐237.2)	214.3 (194.6‐234.0)	Sp & W < S
Triglyceride^a^ (mg/dL)	153.6 (140.1‐167.0)	157.7 (144.3‐171.2)	145.1 (131.6‐158.6)	151.3 (137.8‐164.9)	F < W < S
TProtein (g/dL)	5.83 (5.58‐6.09)	5.75 (5.50‐6.00)	5.76 (5.50‐6.01)	5.84 (5.59‐6.100	S & F < Sp & W
Albumin (g/dL)	3.51 (3.39‐3.62)	3.48 (3.37‐3.59)	3.49 (3.37‐3.60)	3.52 (3.41‐3.63	S & F < W
Globulin (g/dL)	2.33 (2.13‐2.54)	2.68 (2.06‐2.47)	2.28 (2.08‐2.49)	2.33 (2.12‐2.53)	NSD
ALP (IU/L)	720.0 (682.1‐757.8)	721.4 (683.5‐759.3)	697.1 (659.0‐734.2)	700.0 (661.9‐738.1)	F & W < Sp & S
ALT (IU/L)	17.65 (14.58‐20.71)	18.91 (15.84‐21.00)	19.88 (16.81‐22.95)	18.23 (15.16‐21.30)	Sp < S < F
AST (IU/L)	50.48 (46.85‐54.11)	51.08 (47.45‐54.7)	52.78 (49.13‐56.4)	52.05 (48.40‐55.69)	Sp & S < F; Sp < W
GGT (IU/L)	7.71 (5.44‐9.98)	8.12 (5.84‐10.39)	9.00 (6.73‐11.28)	8.20 (5.92‐10.47)	Sp & S & W < F
Phosphorus^a^ (mg/dL)	6.69 (6.49‐6.90)	6.63 (6.42‐6.83)	6.58 (6.37‐6.78)	6.65 (6.44‐6.86)	F < Sp
Sodium (mEq/L)	155.5 (154.8‐156.2)	155.6 (154.9‐156.3)	155.9 (155.2‐156.6)	155.6 (154.9‐156.3)	Sp < F
Chloride (mEq/L)	117.6 (116.6‐118.6)	118.1 (117.1‐119.1)	117.8 (116.9‐118.8)	117.4 (116.4‐118.4)	W & Sp < S; W < F
CO_2_ (mEq/L)	30.23 (29.43‐31.03)	29.88 (29.08‐30.69)	30.41 (29.59‐31.21)	30.71 (29.91‐31.52)	S < F; Sp & S < W

Hb, hemoglobin concentration; RBC, red blood cell count; MCV, mean corpuscular volume; RDW, red blood cell distribution width; platelet, platelet count; MPV, mean platelet volume; Retic, reticulocyte fraction; WBC, total white blood cell count; Abs segs, segmented neutrophil count; Abs lymphs, absolute lymphocyte count; Abs monos, monocyte count; Abs eos, eosinophil counts; ESR60, erythrocyte sedimentation rate at 60 minutes; BUN, blood urea nitrogen; Creat, creatinine; Tbili, total bilirubin; Tprotein, total protein; globulin, total globulins; ALP, alkaline phosphatase; ALT, alanine aminotransferase; AST, aspartate aminotransferase; GGT, gamma‐glutamyl transpeptidase; CK, creatinine kinase; LDH, lactate dehydrogenase; Ca, calcium; P, phosphorus; Na, sodium; K, potassium; Cl, chloride; FIB, fibrinogen.

NSD = Post hoc Šidák pairwise comparisons were not significantly different (*P* < 0.01).

a Analytes for which a significant difference was measured between sex were controlled for by sex.

**Table 4:** Hematologic and serum chemistry analytes (marginal mean, lower 95% confidence limit − higher 95% confidence limit) from 1,084 blood samples of clinically healthy juvenile killer whales (*Orcinus orca*, 3 to <11 years old) for which seasonal differences were detected (N = 23)

Blood analyte	Spring	Summer	Fall	Winter	Šidák Groups (P < 0.01)
Hematology					
Hb (g/dL)	14.5 (14.3‐14.7)	14.7 (14.6‐14.9)	14.6 (14.5‐14.8)	14.5 (14.3‐14.7)	W & Sp < S&F
HCT (%)	42.1 (41.6‐42.6)	42.6 (42.1‐43.1)	42.3 (41.8‐42.8)	41.8 (41.3‐42.3)	W < F & Sp < S
RBC (10^6^/μL)	3.80 (3.72‐3.89)	3.86 (3.77‐3.94)	3.83 (3.74‐3.92)	3.79 (3.70‐3.88)	Sp & W < S & F
MCV (fL)	110.9 (109.8‐112.1)	110.8 (109.7‐112.0)	110.6 (109.4‐111.7)	110.5 (109.3‐111.7)	NSD
Retic^a^ (%)	1.54 (1.40‐1.68)	1.44 (1.30‐1.57)	1.43 (1.29‐1.57)	1.56 (1.42‐1.70)	S & F < Sp & W
WBC (10^3^/μL)	5763.4 (5376.5‐6150.3)	5629.9 (5243.8‐6015.9)	5596.5 (5209.6‐5983.3)	5799.8 (5412.6‐6187.0)	F < Sp & W; S<W
Abs segs (10^3^/μL)	4144.8 (3797.0‐4492.7)	4020.9 (3673.7‐4368.0)	3995.7 (3647.9‐4343.4)	4183.2 (3835.1‐4531.2)	F < Sp & W; S<W
Serum chemistry					
Glucose (mg/dL)	118.9 (116.1‐121.6)	113.6 (110.8‐116.3)	117.8 (115.1‐120.5)	119.6 (116.9‐122.4)	S < Sp & F & W; F < W
BUN (mg/dL)	37.3 (35.0‐39.5)	39.3 (37.0‐41.6)	38.6 (36.3‐40.9)	37.4 (35.1‐39.7)	Sp & W < F < S
Creat (mg/dL)	1.24 (1.13‐1.34)	1.25 (1.14‐1.35)	1.21 (1.11‐1.31)	1.16 (1.06‐1.26)	W < Sp & S & F
Cholesterol (mg/dL)	185.9 (166.8‐205.0)	192.6 (173.6‐211‐7)	190.5 (171.5‐209.6)	187.3 (168.2‐206.4)	Sp & W < S
Triglyceride^a^ (mg/dL)	128.6 (116.7‐140.4)	132.7 (120.9‐144.5)	120.0 (108.2‐131.9)	126.3 (114.5‐138.2)	F < W < S
TProtein (g/dL)	6.17 (5.92‐6.42)	6.09 (5.84‐6.33)	6.09 (5.85‐6.34)	6.18 (5.93‐6.42)	S & F < Sp & W
Albumin (g/dL)	3.41 (3.30‐3.51)	3.38 (3.27‐3.49)	3.39 (3.28‐3.49)	3.42 (3.31‐3.53)	S & F < W
Globulin (g/dL)	2.76 (2.56‐2.95)	2.70 (2.50‐2.89)	2.71 (2.52‐2.90)	2.75 (2.56‐2.94)	NSD
ALP (IU/L)	468.2 (435.4‐501.0)	469.6 (437.0‐502.2)	445.3 (412.5‐478.1)	448.3 (415.4‐481.1)	F & W < Sp & S
ALT (IU/L)	17.45 (14.50‐20.40)	18.71 (15.76‐21.65)	19.68 (16.73‐22.63)	18.03 (15.08‐21.98)	Sp < S < F
AST (IU/L)	45.31 (41.85‐48.77)	45.91 (42.46‐49.36)	47.60 (44.14‐51.06)	46.87 (43.4‐50.33)	Sp & S < F; Sp < W
GGT (IU/L)	10.36 (8.20‐12.52)	10.77 (8.61‐12.92)	11.66 (9.50‐13.82)	10.85 (8.69‐13.01)	Sp & S & W < F
Phosphorus^a^ (mg/dL)	6.29 (6.11‐6.46)	6.22 (6.05‐6.40)	6.17 (5.99‐6.34)	6.25 (6.07‐6.42)	F < Sp
Sodium (mEq/L)	154.7 (154.1‐155.4)	154.8 (154.2‐155.5)	155.1 (154.5‐155.7)	154.8 (154.2‐155.4)	Sp < F
Chloride (mEq/L)	119.3 (118.4‐120.1)	119.8 (118.9‐120.7)	119.5 (118.6‐120.4)	119.1 (118.2‐120.0)	W & Sp < S; W < F
CO_2_ (mEq/L)	28.73 (28.04‐29.42)	28.38 (27.70‐29.07)	28.90 (28.22‐29.60)	29.22 (28.53‐29.91)	S < F, Sp & S < W

Hb, hemoglobin concentration; RBC, red blood cell count; MCV, mean corpuscular volume; RDW, red blood cell distribution width; platelet, platelet count; MPV, mean platelet volume; Retic, reticulocyte fraction; WBC, total white blood cell count; Abs segs, segmented neutrophil count; Abs lymphs, absolute lymphocyte count; Abs monos, monocyte count; Abs eos, eosinophil counts; ESR60, erythrocyte sedimentation rate at 60 minutes; BUN, blood urea nitrogen; Creat, creatinine; Tbili, total bilirubin; Tprotein, total protein; globulin, total globulins; ALP, alkaline phosphatase; ALT, alanine aminotransferase; AST, aspartate aminotransferase; GGT, gamma‐glutamyl transpeptidase; CK, creatinine kinase; LDH, lactate dehydrogenase; Ca, calcium; P, phosphorus; Na, sodium; K, potassium; Cl, chloride; FIB, fibrinogen.

NSD = Post hoc Šidák pairwise comparisons were not significantly different (*P* < 0.01).

a Analytes for which a significant difference was measured between sex were controlled for by sex.

**Table 5:** Hematologic and serum chemistry analytes (marginal mean, lower 95% confidence limit − higher 95% confidence limit) from 794 blood samples of clinically healthy early adult killer whales (*Orcinus orca*, 11 to <21 years old) for which seasonal differences were detected (N = 23)

Blood analyte	Spring	Summer	Fall	Winter	Šidák groups (*P* < 0.01)
Hematology					
Hb (g/dL)	14.7 (14.5‐14.9)	14.9 (14.8‐15.1)	14.8 (14.7‐15.0)	14.7 (14.5‐14.9)	W & Sp & F < S
HCT (%)	43.2 (42.7‐43.7)	43.8 (43.3‐44.3)	43.4 (42.9‐43.9)	43.0 (42.5‐43.5)	W < F & Sp < S
RBC (10^6^/μL)	3.86 (3.77‐3.95)	3.91 (3.83‐4.00)	3.89 (3.80‐3.98)	3.85 (3.76‐3.94)	Sp & W < S & F
MCV (fL)	112.3 (111.1‐113.4)	112.2 (111.0‐113.3)	111.9 (110.7‐113.1)	111.8 (110.7‐113.0)	NSD
Retic^a^ (%)	1.36 (1.22‐1.51)	1.26 (1.12‐1.40)	1.26 (1.12‐1.40)	1.39 (1.25‐1.53)	S & F < Sp & W
WBC (10^3^/μL)	5488.2 (5097.2‐5879.3)	5354.7 (4964.3‐5745.1)	5321.3 (4929.8‐5712.8)	5524.6 (5132.6‐5916.6)	F < Sp & W; S < W
Abs segs (10^3^/μL)	4227.4 (3876.2‐4578.5)	4103.4 (3752.7‐4454.0)	4078.2 (3726.7‐4429.7)	4265.7 (3913.7‐4617.6)	F < Sp & W; S < W
Serum chemistry					
Glucose (mg/dL)	115.4 (112.6‐118.2)	110.1 (107.3‐112.9)	114.4 (111.5‐117.2)	116.2 (113.4‐119.0)	S < Sp & F & W; F<W
BUN (mg/dL)	38.7 (36.4‐41.0)	40.7 (38.4‐43.0)	40.0 (37.7‐41.2)	38.9 (36.6‐41.2)	Sp & W < F < S
Creat (mg/dL)	1.69 (1.59‐1.79)	1.70 (1.60‐1.80)	1.67 (1.57‐1.77)	1.61 (1.51‐1.71)	W < Sp & S & F
Cholesterol (mg/dL)	194.6 (175.5‐213.8)	201.3 (182‐220.5)	199.2 (180.1‐218.4)	196.0 (176.8‐215.2)	Sp & W < S
Triglyceride^a^ (mg/dL)	141.1 (129.1‐153.2)	145.3 (133.2‐157.3)	132.6 (120.5‐144.7)	138.9 (126.8‐151.0)	F < W < S
TProtein (g/dL)	6.92 (6.67‐7.17)	6.83 (6.59‐7.08)	6.84 (6.59‐7.09)	6.93 (6.68‐7.18)	S & F < Sp & W
Albumin (g/dL)	3.61 (3.50‐3.71)	3.58 (3.47‐3.69)	3.59 (3.48‐3.69)	3.62 (3.51‐3.73)	S & F < W
Globulin (g/dL)	3.30 (3.11‐3.50)	3.23 (3.04‐3.42)	3.25 (3.06‐3.45)	3.30 (3.10‐3.49)	NSD
ALP (IU/L)	245.7 (212.2‐279.2)	247.1 (213.7‐280.5)	222.8 (189.2‐256.4)	225.8 (192.1‐259.5)	F & W < Sp & S
ALT (IU/L)	14.33 (11.36‐17.29)	15.58 (12.62‐18.55)	16.55 (13.59‐19.53)	14.91 (11.94‐17.88)	Sp < S < F
AST (IU/L)	40.06 (36.57‐43.54)	40.66 (37.18‐44.14)	42.34 (38.86‐45.83)	41.62 (38.13‐45.11)	Sp & S < F; Sp < W
GGT (IU/L)	9.97 (7.80‐12.15)	10.38 (8.20‐12.56)	11.27 (9.09‐13.45)	10.47 (8.28‐12.65)	Sp & S & W < F
Phosphorus^a^ (mg/dL)	5.55 (5.37‐5.73)	5.48 (5.31‐5.66)	5.43 (5.25‐5.61)	5.51 (5.33‐5.69)	F < Sp
Sodium (mEq/L)	153.9 (153.2‐154.5)	154.0 (153.3‐154.6)	154.2 (153.6‐154.9)	153.9 (153.4‐154.6)	Sp < F
Chloride (mEq/L)	120.2 (119.3‐121.1)	120.7 (119.8‐121.6)	120.4 (119.5‐121.3)	119.9 (119.0‐120.9)	W & Sp < S; W < F
CO_2_ (mEq/L)	27.61 (26.91‐28.32)	27.26 (26.56‐28.00)	27.79 (27.08‐28.49)	28.10 (27.39‐28.81)	S < F; Sp & S < W

Hb, hemoglobin concentration; RBC, red blood cell count; MCV, mean corpuscular volume; RDW, red blood cell distribution width; platelet, platelet count; MPV, mean platelet volume; Retic, reticulocyte fraction; WBC, total white blood cell count; Abs segs, segmented neutrophil count; Abs lymphs, absolute lymphocyte count; Abs monos, monocyte count; Abs eos, eosinophil counts; ESR60, erythrocyte sedimentation rate at 60 minutes; BUN, blood urea nitrogen; Creat, creatinine; Tbili, total bilirubin; Tprotein, total protein; globulin, total globulins; ALP, alkaline phosphatase; ALT, alanine aminotransferase; AST, aspartate aminotransferase; GGT, gamma‐glutamyl transpeptidase; CK, creatinine kinase; LDH, lactate dehydrogenase; Ca, calcium; P, phosphorus; Na, sodium; K, potassium; Cl, chloride; FIB, fibrinogen.

NSD = Post hoc Šidák pairwise comparisons were not significantly different (*P* < 0.01).

a Analytes for which a significant difference was measured between sex were controlled for by sex.

**Table 6:** Hematologic and serum chemistry analytes (marginal mean, lower 95% confidence limit − higher 99% confidence limit) from 476 blood samples of clinically healthy adult killer whales (*Orcinus orca*, 21 to <31 years old) for which seasonal differences were detected (N = 23)

Blood analyte	Spring	Summer	Fall	Winter	Šidák Groups (P < 0.01)
Hematology					
Hb (g/dL)	15.0 (14.8‐15.2)	15.2 (15.1‐15.4)	15.2 (15.1‐15.4)	15.0 (14.8‐15.2)	NSD
HCT (%)	44.7 (44.2‐45.25)	45.3 (4.8‐45.8)	44.9 (44.4‐45.4)	44.5 (43.9‐45.0)	W<F; Sp < S
RBC (10^6^/μL)	4.00 (3.90‐4.08)	4.04 (3.95‐4.13)	4.02 (3.93‐4.11)	3.98 (3.89‐4.07)	Sp & W < S & F
MCV (FL)	112.4 (111.2‐113.6)	112.3 (111.1‐113.5)	112.1 (110.9‐113.3)	112.0 (110.8‐113.2)	NSD
Retic^a^ (%)	1.10 (0.95‐1.26)	1.00 (0.84‐1.16)	1.0 (0.84‐1.15)	1.13 (0.97‐1.29)	S & F < Sp & W
WBC (10^3^/μL)	5012.0 (4602.1‐5286.9)	4878.4 (4469.9‐5286.9)	4845.0 (4435.9‐5254.1)	5048.4 (4638.2‐5458.5)	F < Sp & W; S < W
Abs segs (10^3^/μL)	4064.5 (3698.0‐4431.0)	3940.5 (3575.1‐4305.9)	3915.3 (3549.5‐4281.1)	4102.8 (3736.2‐4469.5)	F < Sp & W; S < W
Serum chemistry					
Glucose (mg/dL)	112.1 (109.0‐115.3)	106.9 (103.7‐110.0)	111.1 (108.0‐114.2)	112.9 (109.8‐116.0)	S < Sp & F & W; F < W
BUN (mg/dL)	40.1 (37.7‐42.5)	42.1 (39.8‐44.5)	41.4 (39.0‐43.8)	40.3 (37.9‐42.6)	Sp & W < F < S
Creat (mg/dL)	1.90 (1.79‐2.00)	1.91 (1.80‐2.01)	1.87 (1.77‐1.97)	1.82 (1.71‐1.92)	W < Sp & S & F
Cholesterol (mg/dL)	203.5 (184.0‐223.1)	210.2 (190.7‐229.7)	208.1 (188.6‐227.7)	204.9 (185.3‐224.5)	Sp & W < S
Triglyceride^a^ (mg/dL)	142.1 (129.1‐155.2)	146.3 (133.2‐159.3)	133.6 (120.6‐146.7)	139.9 (126.8‐153.0)	F < W < S
TProtein (g/dL)	7.17 (6.91‐7.42)	7.08 (6.83‐7.33)	7.09 (6.83‐7.34)	7.17 (6.91‐7.42)	S & F < Sp & W
Albumin (g/dL)	3.72 (3.60‐3.83)	3.69 (3.58‐3.80)	3.70 (3.58‐3.81)	3.73 (3.62‐3.84)	S & F < W
Globulin (g/dL)	3.46 (3.25‐3.66)	3.39 (3.19‐3.60)	3.41 (3.20‐3.61)	3.45 (3.25‐3.65)	NSD
ALP (IU/L)	139.0 (102.3‐175.6)	140.4 (103.9‐176.8)	116.1 (79.5‐152.6)	119.0 (82.3‐155.7)	F & W < Sp & S
ALT (IU/L)	13.79 (10.74‐16.83)	15.04 (12.01‐18.08)	16.02 (12.98‐19.06)	14.37 (11.32‐17.41)	Sp < S < F
AST (IU/L)	40.51 (36.91‐44.11)	41.11 (37.53‐44.71)	42.81 (39.21‐46.40)	42.08 (38.48‐45.68)	Sp & S < F; Sp < W
GGT (IU/L)	7.23 (4.97‐9.49)	7.64 (5.38‐9.89)	8.52 (6.27‐10.78)	7.72 (5.46‐9.98)	Sp & S & W < F
Phosphorus^a^ (mg/dL)	5.30 (5.11‐5.01)	5.24 (5.04‐5.43)	5.19 (4.99‐5.38)	5.26 (5.07‐5.46)	F < Sp
Sodium (mEq/L)	152.7 (152.0‐153.4)	152.8 (152.1‐153.5)	153.1 (152.4‐153.8)	152.8 (152.1‐153.5)	Sp < F
Chloride (mEq/L)	120.2 (119.1‐121.1)	120.7 (119.7‐121.6)	120.4 (119.4‐121.4)	119.9 (119.0‐120.9)	W & Sp < S; W < F
CO_2_ (mEq/L)	27.45 (26.68‐28.23)	27.11 (26.34‐27.88)	27.63 (27.86‐28.40)	27.95 (27.17‐28.72	S < F; Sp & S < W

Hb, hemoglobin concentration; RBC, red blood cell count; MCV, mean corpuscular volume; RDW, red blood cell distribution width; platelet, platelet count; MPV, mean platelet volume; Retic, reticulocyte fraction; WBC, total white blood cell count; Abs segs, segmented neutrophil count; Abs lymphs, absolute lymphocyte count; Abs monos, monocyte count; Abs eos, eosinophil counts; ESR60, erythrocyte sedimentation rate at 60 minutes; BUN, blood urea nitrogen; Creat, creatinine; Tbili, total bilirubin; Tprotein, total protein; globulin, total globulins; ALP, alkaline phosphatase; ALT, alanine aminotransferase; AST, aspartate aminotransferase; GGT, gamma‐glutamyl transpeptidase; CK, creatinine kinase; LDH, lactate dehydrogenase; Ca, calcium; P, phosphorus; Na, sodium; K, potassium; Cl, chloride; FIB, fibrinogen.

NSD = Post hoc Šidák pairwise comparisons were not significantly different (*P* < 0.01).

a Analytes for which a significant difference was measured between sex were controlled for by sex.

**Table 7:** Hematologic and serum chemistry analytes (marginal mean, lower 95% confidence limit − higher 95% confidence limit) from 248 blood samples of clinically healthy aged adult killer whales (*Orcinus orca*, 31+ years old) for which seasonal differences were detected (N = 23)

Blood analyte	Spring	Summer	Fall	Winter	Šidák groups (*P* < 0.01)
Hematology					
Hb (g/dL)	15.1 (14.9‐15.3)	15.3 (15.1‐15.5)	15.2 (15.0‐15.4)	15.1 (14.9‐15.3)	NSD
HCT (%)	45.3 (44.7‐45.9)	45.9 (45.3‐46.5)	45.5 (44.9‐46.1)	45.1 (44.5‐45.6)	W < F; Sp < S
RBC (10^6^/μL)	4.03 (3.94‐4.13)	4.08 (3.99‐4.18)	4.06 (3.97‐4.16)	4.02 (3.92‐4.12)	Sp & W < S & F
MCV (FL)	113.0 (111.7‐114.3)	112.9 (111.6‐114.2)	112.7 (111.4‐114.0)	112.6 (111.3‐113.9)	NSD
Retic^a^ (%)	1.03 (0.84‐1.23)	0.93 (0.74‐1.12)	0.93 (0.748‐1.12)	1.06 (0.87‐1.25)	S & F < Sp & W
WBC (10^3^/μL)	4839.3 (4385.7‐5292.9)	4705.7 (4253.4‐5158.1)	4672.3 (4220.5‐5124.1)	4875.7 (4422.7‐5328.7)	F < Sp & W; S < W
Abs segs (10^3^/μL)	4019.4 (3616.9‐4421.9)	3895.4 (3494.0‐4296.8)	3870.2 (3469.1‐4271.3)	4057.7 (3655.7‐4459.7)	F < Sp & W; S < W
Serum chemistry					
Glucose (mg/dL)	106.4 (102.6‐110.3)	101.2 (97.4‐104.9)	105.4 (101.6‐109.2)	107.2 (103.4‐111.0)	S < Sp & F & W; F<W
BUN (mg/dL)	41.3 (38.8‐43.8)	43.4 (40.8‐45.9)	42.6 (40.1‐45.1)	41.5 (39.0‐44.0)	Sp & W < F < S
Creat (mg/dL)	1.99 (1.88‐2.11)	2.00 (1.89‐2.11)	1.97 (1.86‐2.08)	1.92 (1.80‐2.03)	W < Sp & S & F
Cholesterol (mg/dL)	220.5 (200.0‐241.0)	227.2 (206.7‐247.6)	225.1 (204.6‐245.5)	221.9 (201.4‐242.3)	Sp & W < S
Triglyceride^a^ (mg/dL)	155.4 (139.9‐170.9)	159.5 (144.0‐175.0)	146.9 (131.4‐162.3)	153.1 (137.6‐168.7)	F < W < S
TProtein (g/dL)	7.53 (7.26‐7.80)	7.45 (7.18‐7.72)	7.545 (7.19‐7.72)	7.53 (7.27‐7.81)	S & F < Sp & W
Albumin (g/dL)	3.88 (3.77‐4.00)	3.86 (3.74‐3.97)	3.86 (3.75‐4.00)	3.90 (3.78‐4.01)	S & F < W
Globulin (g/dL)	3.64 (3.42‐3.86)	3.58 (3.36‐3.80)	3.59 (3.37‐3.81)	3.63 (3.41‐3.85)	NSD
ALP (IU/L)	93.0 (49.3‐136.8)	94.4 (50.8‐138.0)	70.1 (26.7‐113.6)	73.1 (29.4‐116.7)	F & W < Sp & S
ALT (IU/L)	13.65 (10.42‐16.87)	14.90 (11.68‐18.12)	15.88 (12.66‐19.09)	14.23 (11.00‐17.45)	Sp < S < F
AST (IU/L)	41.06 (37.20‐44.93)	41.67 (37.81‐45.53)	43.39 (39.51‐47.21)	42.63 (38.77‐46.49)	Sp & S < F; Sp < W
GGT (IU/L)	3.73 (1.15‐6.31)	4.14 (1.56‐6.71)	5.02 (2.45‐7.59)	4.22 (1.64‐6.79)	Sp & S & W < F
Phosphorus^a^ (mg/dL)	5.56 (5.32‐5.80)	5.49 (5.25‐5.74)	5.44 (5.20‐5.69)	5.52 (5.28‐5.76)	F < Sp
Sodium (mEq/L)	151.4 (150.6‐152.2)	151.6 (150.8‐152.4)	151.8 (151.0‐152.6)	151.5 (150.7‐152.3)	Sp < F
Chloride (mEq/L)	119.3 (118.2‐120.3)	119.8 (118.7‐120.8)	119.5 (118.4‐120.6)	119.0 (118.0‐120.1)	W & Sp < S; W < F
CO_2_ (mEq/L)	27.51 (26.59‐28.44)	27.16 (26.24‐28.09)	27.69 (26.77‐28.61)	28.00 (27.08‐28.93)	S < F; Sp & S < W

Hb, hemoglobin concentration; RBC, red blood cell count; MCV, mean corpuscular volume; RDW, red blood cell distribution width; platelet, platelet count; MPV, mean platelet volume; Retic, reticulocyte fraction; WBC, total white blood cell count; Abs segs, segmented neutrophil count; Abs lymphs, absolute lymphocyte count; Abs monos, monocyte count; Abs eos, eosinophil counts; ESR60, erythrocyte sedimentation rate at 60 minutes; BUN, blood urea nitrogen; Creat, creatinine; Tbili, total bilirubin; Tprotein, total protein; globulin, total globulins; ALP, alkaline phosphatase; ALT, alanine aminotransferase; AST, aspartate aminotransferase; GGT, gamma‐glutamyl transpeptidase; CK, creatinine kinase; LDH, lactate dehydrogenase; Ca, calcium; P, phosphorus; Na, sodium; K, potassium; Cl, chloride; FIB, fibrinogen.

NSD = Post hoc Šidák pairwise comparisons were not significantly different (*P* < 0.01).

a Analytes for which a significant difference was measured between sex were controlled for by sex.

The impact of season on each of the remaining 20 analytes followed identical trends in calf, juvenile, early adult, adult, and aged whales (Tables 3, 4, 5, 6, 7). The analytes most often peaked in summer (N = 8: HCT, RBC, BUN, Creat, cholesterol, triglycerides, ALP, and chloride), followed by winter (N = 7: Retic, WBC, Abs segs, glucose, Tprotein, albumin, and CO_2_), fall (N = 4: ALT, AST, GGT, and sodium) and spring (N = 1: phosphorus). Conversely, the analytes that least often dipped in summer (N = 4: glucose, TProtein, albumin, CO_2_) and winter (N = 4: HCT, RBC, Creat, chloride), followed by fall (N = 6: Retic, WBC, Abs segs, triglycerides, ALP, phosphorus) and spring (N = 6: BUN, Cholesterol, ALT, AST, GGT, sodium). Corresponding peaks and dips of analytes were more commonly detected during opposite (N = 14) than during adjacent seasons (N = 6).

## 4 DISCUSSION

Of the variables measured, age had the biggest influence on blood analytes. All 39 blood analytes surveyed in this study were affected by age. Additionally, season and sex influenced 59% and 10% of the analytes, respectively. Many, but not all, of these effects on baseline hematologic and plasma biochemical values had been previously recognized in other cetacean species.^8^, ^9^, ^10^, ^11^, ^12^, ^13^, ^14^, ^15^, ^16^, ^17^, ^18^, ^19^, ^20^, ^21^ This report is based on a larger sample size than any of the previously published reports on cetacean RIs, and samples were selected using stringent inclusion criteria, which could account for the high analyte percentages recognized as being significantly affected by these variables.

Contrary to previous publications where sex was shown to be a potentially important influencer of hematologic and biochemical variables in marine mammals,^12^, ^20^, ^21^ we detected only four (10%) analytes (RDW, MPV, triglycerides, and phosphorus) that differed between male and female killer whales. Male killer whales had higher (*P* = 0.015) triglyceride levels than female killer whales, whereas free‐ranging female dolphins had higher triglyceride levels than male dolphins.^12^ The sex differences between RDW, MPV, and phosphorus observed in this study have not been reported in other cetacean studies. Our findings were similar to earlier killer whale studies, which showed that Hb concentrations, HCTs, RBCs, and WBCs did not differ between male and female killer whales.^23^ However, many sex differences that were previously described in bottlenose dolphins (HCT, RBC, WBC, Abs lymphs, Abs eos, ESR, glucose, BUN, creatinine, cholesterol, total protein, albumin, GGT, CK, sodium, potassium, and iron) and belugas (Hb, RBC, MCV, WBC, Abs segs, ALT, and potassium) were not recognized in this population of killer whales.^20^, ^21^ These sex differences had been partially attributed to pregnancy or shifts in reproductive hormones.^20^, ^21^ Since the reproductive status of the female killer whales enrolled in this study was closely monitored, we were able to exclude samples from females that were pseudopregnant, pregnant, or in early lactation. These exclusions appeared to have eliminated sex biases, thereby supporting that sex differences observed in previous studies could be attributed to pregnancy, pseudopregnancy, and early lactation.^20^, ^21^ Since we did not eliminate females who were experiencing regular estrous cycles or males who had seasonal peaks of serum testosterone,^29^, ^30^ we have also provided evidence that these physiologic events do not result in hematologic and serum biochemical differences between male and female killer whales as has been proposed.^20^, ^21^

Creatinine levels have been reported to be higher in male dolphins and belugas, which was attributed to the increased muscle mass of male specimens.^15^, ^16^, ^19^ Male killer whales over 20 years of age (adult and aged groups) are markedly larger than female killer whales^31^and Cornell^23^ did note a similar increase in creatinine levels in large adult male killer whales compared with smaller ones.^23^ However, in our results, while creatinine differences clearly occurred with age and season, we did not detect differences between males and females.

Most analytes (33/39; 85%) showed a progressive upward or downward trend with age, as would be expected with the true physiologic changes associated with aging. The number of differences detected between analytes decreased as killer whales transitioned between age groups, with the least number of differences detected between the adult and aged groups, suggesting that the greatest physiologic changes happen during the years of development and growth.

Aging killer whales showed evidence of immune senescence, rather than a state of chronic inflammation reported in aging dolphins.^18^ While killer whale globulin levels did increase with age, WBC, Abs segs, Abs lymphs, Abs monos, Abs eos, ESR60, and FIB decreased progressively. Cornell^23^ noted a similar decrease in WBCs with age in killer whales. In humans and other mammals, advanced age alters both the innate and adaptive immune systems.^32^, ^33^ Immune senescence starts as the thymus shrinks, eventually resulting in a decline of naïve T cells. As T‐cell receptor repertoire diversity narrows and B cell‐induced antibody affinity declines with aging, lymphoid lineage decreases, and myeloid lineage hematopoietic stem cells become proportionally more dominant. In addition, many of the myeloid‐derived, innate effector functions including phagocytosis are downregulated by aging as well. In humans, immune senescence leads to both decreased responses to acute infections and poor development of immunologic memory.^32^, ^33^ These and other age‐related changes involving the immune system predispose the elderly to infectious diseases, cardiovascular diseases, atherosclerosis, autoimmune diseases, neoplasia, anemia, degenerative diseases including metabolic syndrome, and organ failure, such as renal failure. A review of morbidity and mortality data for killer whales would be needed to determine whether immune senescence predisposes killer whales to these syndromes as well.

Red blood cell parameters (Hb, HCT, RBC, and RDW) of killer whales increased progressively with age. The MCHC value for the red cells decreased, and while this was. statistically significant, the difference was subtle and might be physiologically insignificant. Erythrocyte parameters reflect the capacity to transport oxygen to ensure metabolic processes, cell survival, and typically increase with age in fish, birds, turtles, and various other mammals.^34^, ^35^, ^36^, ^37^, ^38^, ^39^, ^40^ Red cell parameters similarly increased in beluga whales housed at SeaWorld parks.^20^ The findings in that beluga whale study and the killer whale findings herein are in contrast with the progressive anemia that has been reported in aging dolphins^18^ and a population of managed beluga whales in Taiwan.^21^ In humans, anemia in elderly patients can be divided into three subtypes: anemias with a nutrient deficiency (eg, folate), anemias without a nutrient deficiency (eg, anemia of chronic inflammation), and unexplained anemias. Anemia of chronic inflammation is the most prevalent subtype in elderly people, but the increase in chronic inflammation, which was reported in aging dolphins in one facility,^18^ was not detected in these killer whales. Alternatively, the anemia reported in some aging cetaceans might not be present in the killer whales of this study, as they were supplemented with a multivitamin containing folate.

Platelet counts decreased in the aging killer whales, while the MPV progressively increased. Reduced platelet counts have been reported in some beluga whales.^21^ Megakaryocytes are the precursor cells of platelets, and dolphins, unlike most other mammals, have megakaryocytes in both bone marrow and spleen, a proposed physiologic adaptation of diving mammals.^41^ Considering dolphins and, presumably, killer whales have this extra capability for generating platelets, the decrease in platelet counts might reflect a decrease in the platelet lifespan.^42^ Alternatively, the decreased platelet count could be a function of the previously mentioned immune senescence, as platelets are increasingly recognized as immune sentinels, being both modulators and effectors of the immune system.^43^ The MPV increased in aging dolphins^18^ and killer whales. The MPV represents platelet activity and is most commonly used to discriminate causes of thrombocytopenia.^44^ If the platelet count of a killer whale were to drop below the physiologic ranges established in this study, along with a rise in the MPV, diseases such as autoimmune thrombocytopenic purpura, preeclampsia, disseminated intravascular coagulation, and sepsis would need to be considered.

BUN and creatinine increased progressively with age, as had been previously reported in killer whales.^23^ These parameters are usually interpreted as renal clearance assays. The whales enrolled in this study were clinically healthy; however, subclinical renal perfusion or clearance might have been decreased in older killer whales. Alternatively, blood urea is a breakdown product of protein intake, and increased BUN could reflect a higher food intake by the older and larger animals. Creatinine is a breakdown product of creatine phosphate by creatinine kinase in muscle and is, in healthy animals, a reflection of muscle mass.^45^ However, the decrease in creatinine and, by extrapolation, in muscle mass reported in some other cetacean populations^21^ does not occur in this population of killer whales. Therefore, the combined elevation of both BUN and creatinine with age supports decreasing renal perfusion or clearance in aged killer whales. Considering that the red blood cell parameters, Hb, HCT, and RBC, also progressively increased with age, prerenal causes for decreased renal clearance need to be considered.

The liver‐associated analytes, ALP, ALT, AST, and GGT, decreased progressively with age,^45^ while total bilirubin increased. AST and ALT are enzymes present in both cytoplasm and mitochondria of liver, heart, skeletal muscle, and kidney cells, but both enzymes are predominantly found in the liver.^1^ Bilirubin is typically interpreted as a hepatic clearance assay. GGT was low in killer whale calves, peaked in juveniles, and then progressively decreased as the killer whales aged. GGT is an enzyme found in high concentration in the liver and kidney, but the GGT enzyme present in the serum is mainly from the hepatobiliary system.^1^ The decrease in hepatocellular enzymes and the concurrent increase in total bilirubin could indicate decreased hepatocellular expression of these enzymes and decreased liver clearance capability in aging killer whales. Similar trends were reported in managed and wild beluga whales.^13^, ^20^, ^21^ However, albumin, which is synthesized by the liver, was higher in the older killer whales of this study.

Young killer whales had higher ALP and calcium levels. Changes in alkaline phosphatase levels can indicate both liver and bone disorders and bone metabolism since it is primarily present in these tissues.^1^ The higher ALP activity and plasma calcium concentration in younger animals are usually attributed to active bone growth.^10^, ^11^, ^15^, ^20^, ^21^ Sodium and potassium concentrations decreased with age, which could imply that changes in the ability of electrolyte and fluid management exist with maturation.^10^ Similarly, glucose levels were higher in younger killer whales as has been previously reported in managed killer whales,^23^ free‐ranging dolphins,^16^ managed beluga whales,^20^, ^21^ but not managed dolphins at one facility.^15^

Seasonal variation (23 of 39 analytes) was detected in more analytes in this population of killer whales than in any of the previous studies in which the effect of season on blood analytes of cetaceans was explored.^14^, ^19^, ^20^, ^21^ The killer whales in this study were housed at approximately 14°C and fed a consistent diet year‐round, whereas free‐ranging killer whales frequently inhabit places with varying temperatures in search of adequate prey resources.^46^ Some of the annual circadian patterns, such as food availability, changes in diving patterns, body condition, and water temperature, to which seasonal variation was attributed in free‐ranging cetaceans, are eliminated in this killer whale population. Instead, the high degree of seasonal variation that we detected with high confidence in the dataset could largely be due to diffuse seasonal cycling patterns and the seasonality of sex hormones in killer whales.^29^, ^30^, ^47^ Alternatively, photoperiods and subsequent endocrine fluctuations that are not related to reproduction, could be the primary drivers. This is supported by the fact that the seasonal impact followed identical trends in the killer whale calf group for most analytes in which seasonality was detected, including sexually immature calves.

The detected seasonal variation in all but one (WBC) of the blood analytes had previously been recognized in other cetacean species.^14^, ^19^, ^20^, ^21^ Seasonal variation is surprisingly similar between managed (in this report, and others^20^, ^21^) and free‐ranging cetaceans.^14^, ^19^ MCHC was the only analyte with detectable seasonal variability in all free‐ranging populations only. No seasonal variability was detected exclusively in managed cetacean populations. Total protein,^14^, ^19^, ^20^, ^21^ HCT, 14, 19, 20 RBC,^14^, ^20^, ^21^ creatinine,^14^, ^19^, ^20^ and chloride^19^, ^20^, ^21^ showed consistent seasonal patterns in managed and free‐ranging cetaceans. In all of these populations, HCT, RBC, creatinine, and chloride peaked around the summer months. Erythropoiesis is known to vary by season^48^, ^49^ and cetacean erythropoietic activity peaks in the summer months.^14^, ^20^ Creatinine levels could be caused by muscle volume and activity, and dietary protein intake, to a lesser degree.^1^ It appears that either muscle mass or metabolism fluctuates by season, even in populations with a consistent diet, food intake, and activity level.

Established RIs are important tools used in the care and diagnostics of managed and stranded animals. RIs are also used for population health investigations. Wild killer whale populations are exposed to stressors such as food limitations and the persistent presence of organochlorines, which are suspected of being immunomodulatory.^50^, ^51^, ^52^ The availability of established RIs for killer whales and an understanding of the variables affected by physiologic stimuli will be necessary for investigations into the impact of these stressors on killer whales. The subjects in this study were clinically healthy killer whales, free of parasites, free of immunomodulatory organochlorine pollutants, and fed a consistent and adequate diet. In addition, samples were collected without cortisol‐induced artifacts as would be triggered by the capture of a free‐ranging cetacean. Therefore, it is expected that free‐ranging killer whale blood analyte levels would deviate from those reported here.^19^, ^20^, ^53^ However, the sample numbers needed to establish RIs cannot be obtained from free‐ranging killer whales. This dataset and the trends described herein provide a unique and invaluable tool for evaluating free‐ranging killer whales, especially when extrapolated and interpreted by experienced cetacean clinicians.

## DISCLOSURE

The authors have indicated that they have no affiliations or financial involvement with any organization or entity with a financial interest in, or in financial competition with, the subject matter or materials discussed in this article.
